# Differential Effects of 17,18-EEQ and 19,20-EDP Combined with Soluble Epoxide Hydrolase Inhibitor *t*-TUCB on Diet-Induced Obesity in Mice

**DOI:** 10.3390/ijms22158267

**Published:** 2021-07-31

**Authors:** Yang Yang, Xinyun Xu, Haoying Wu, Jun Yang, Jiangang Chen, Christophe Morisseau, Bruce D. Hammock, Ahmed Bettaieb, Ling Zhao

**Affiliations:** 1 Department of Nutrition, University of Tennessee, Knoxville, TN 37996, USA; yyang100@vols.utk.edu (Y.Y.); xxu28@vols.utk.edu (X.X.); hwu26@vols.utk.edu (H.W.); abettaie@utk.edu (A.B.); 2 Department of Entomology and Nematology, and Comprehensive Cancer Center, University of California, Davis, CA 95616, USA; junyang@ucdavis.edu (J.Y.); chmorisseau@ucdavis.edu (C.M.); bdhammock@ucdavis.edu (B.D.H.); 3 Department of Public Health, University of Tennessee, Knoxville, TN 37996, USA; jchen38@utk.edu; 4 Graduate School of Genome Science and Technology, University of Tennessee, Knoxville, TN 37996, USA

**Keywords:** soluble epoxide hydrolase, soluble epoxide hydrolase inhibitor, *t*-TUCB, 19,20-EDP, 17,18-EEQ, brown adipose tissue, brown adipogenesis

## Abstract

17,18-Epoxyeicosatetraenoic acid (17,18-EEQ) and 19,20-epoxydocosapentaenoic acid (19,20-EDP) are bioactive epoxides produced from n-3 polyunsaturated fatty acid eicosapentaenoic acid and docosahexaenoic acid, respectively. However, these epoxides are quickly metabolized into less active diols by soluble epoxide hydrolase (sEH). We have previously demonstrated that an sEH inhibitor, *t*-TUCB, decreased serum triglycerides (TG) and increased lipid metabolic protein expression in the brown adipose tissue (BAT) of diet-induced obese mice. This study investigates the preventive effects of *t*-TUCB (T) alone or combined with 19,20-EDP (T + EDP) or 17,18-EEQ (T + EEQ) on BAT activation in the development of diet-induced obesity and metabolic disorders via osmotic minipump delivery in mice. Both T + EDP and T + EEQ groups showed significant improvement in fasting glucose, serum triglycerides, and higher core body temperature, whereas heat production was only significantly increased in the T + EEQ group. Moreover, both the T + EDP and T + EEQ groups showed less lipid accumulation in the BAT. Although UCP1 expression was not changed, PGC1α expression was increased in all three treated groups. In contrast, the expression of CPT1A and CPT1B, which are responsible for the rate-limiting step for fatty acid oxidation, was only increased in the T + EDP and T + EEQ groups. Interestingly, as a fatty acid transporter, CD36 expression was only increased in the T + EEQ group. Furthermore, both the T + EDP and T + EEQ groups showed decreased inflammatory NFκB signaling in the BAT. Our results suggest that 17,18-EEQ or 19,20-EDP combined with *t*-TUCB may prevent high-fat diet-induced metabolic disorders, in part through increased thermogenesis, upregulating lipid metabolic protein expression, and decreasing inflammation in the BAT.

## 1. Introduction

Obesity remains one of the biggest public health challenges worldwide [1]. Obesity is associated with various comorbidities, including type 2 diabetes, dyslipidemia, and cardiovascular diseases, leading to a shorter lifespan and higher medical costs [2,3]. Recent studies have indicated that obesity is also associated with a high prevalence and severity of COVID-19, an infectious disease caused by the coronavirus SARS-CoV-2 [4,5]. Brown adipose tissue (BAT) has emerged as a promising target for obesity treatment and prevention due to its ability to increase energy expenditure through nonshivering thermogenesis [6,7,8,9]. BAT activation by cold exposure has been reported to improve glucose and lipid metabolism in rodents and humans due to its thermogenic function [10,11,12]. Therefore, strategies that enhance BAT activation and improve BAT function may confer beneficial diet-induced obesity and associated metabolic comorbidities.

N-3 epoxy fatty acids (EpFAs) are bioactive metabolites generated by cytochrome P-450 (CYP450) epoxygenases from n-3 polyunsaturated fatty acids (PUFAs) [13,14]. 17,18-Epoxyeicosatetraenoic acids (17,18-EEQ) and 19,20-epoxydocosapentaenoic acids (19,20-EDP) are the major EpFAs derived from eicosapentaenoic acid (EPA) and docosahexaenoic acid (DHA), respectively. These EpFAs modulate angiogenesis, vascular dilation, inflammation, and cell growth and differentiation, and are implicated in tumor growth and metastasis, cardiovascular disease, diabetes, metabolic syndromes, and pain [15,16,17]. Because these epoxides are unstable and quickly metabolized to less active diols by soluble epoxide hydrolase (sEH), a cytosolic enzyme encoded by the *Ephx2* gene [14], many of the beneficial effects of these EpFAs were potentiated by co-administration of an sEH inhibitor [14,16,17].

Recent studies indicated that higher sEH activity was associated with obesity and metabolic diseases in rodents [18]. Further, sEH expression in subcutaneous adipose tissue of obese adults was significantly higher than that of lean subjects [19]. Therefore, sEH ablation or inhibition has become a promising strategy to combat obesity and associated metabolic disorders by stabilizing endogenous EpFAs. For example, a potent sEH inhibitor, *trans*-4-{4-[3-(4-trifluoromethoxyphenyl)-ureido]cyclohexyloxy}benzoic acid (*t*-TUCB), was reported to improve metabolic health in rodents [20,21]. It is worth noting that in some disease models, sEH inhibitor alone did not show strong beneficial effects unless it was combined with an EpFA [22] or enriched tissue levels of n-3 PUFAs [23]. 

We have previously demonstrated that *t*-TUCB promoted brown adipogenesis in vitro, decreased the serum triglycerides (TG) levels, and increased the protein expression of lipid metabolic genes in the BAT of diet-induced obese mice [24]. In this study, we further investigated the preventive effects of *t*-TUCB (T) alone or combined with 19,20-EDP (T + EDP) or 17,18-EEQ (T + EEQ) on BAT activation in the development of diet-induced obesity and associated metabolic disorders. 

## 2. Results

### 2.1. 17,18-EEQ or 19,20-EDP Combined with t-TUCB Differentially Affected Bodyweight in Diet-Induced Obesity

To investigate whether treatments of *t*-TUCB alone or combined either with 17,18-EEQ or 19,20-EDP are beneficial in preventing the development of diet-induced obesity, osmotic minipumps filled with a vehicle control, *t*-TUCB (T) alone, or T combined with 19,20-EDP (T + EDP) or 17,18-EEQ (T + EEQ) were implanted into the subcutaneous compartment on top of the interscapular BAT (iBAT) of C57BL/6J male mice fed a high-fat diet (45% kcal from fat). In addition, we set up another two groups (*n* = 5 per group) of mice fed either a low-fat diet (10% kcal from fat) or a high-fat diet (45% kcal from fat) for 6 weeks without osmotic minipumps implantation as the non-implantation controls (Supplemental Figure S1).

**Figure 1:**
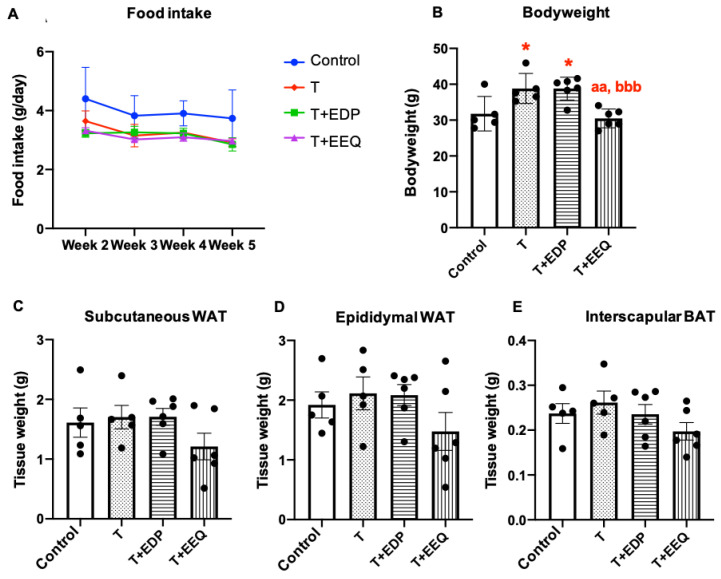
17,18-EEQ or 19,20-EDP combined with *t*-TUCB delivered via mini osmotic pump affected bodyweight differentially in diet-induced obesity. Male C57BL/6J mice fed with a high-fat diet (45% kcal from fat) were treated with *t*-TUCB (3 mg/kg/day) alone or combined with 19,20-EDP (0.05 mg/kg/day) (T + EDP) or 17,18-EEQ (0.05 mg/kg/day) (T + EEQ) by osmotic minipump implantation for 6 weeks, as described in the methods section. Average daily food intake after implantation (**A**), bodyweight (**B**), subcutaneous WAT (**C**), epididymal WAT (**D**), and interscapular BAT weight (**E**) at the end of 6 weeks are shown. Data = the mean ± SEM (*n* = 5–6). * = *p* < 0.05 compared to the controls. ^aa^ = *p* < 0.01 compared to the T group. ^bbb^ = *p* < 0.001 compared to the T + EDP group.

After 6 weeks of treatment, *t*-TUCB alone or combined with either 19,20-EDP or 17,18-EEQ did not change the food intake and individual fat pad mass, including subcutaneous and epididymal WAT and iBAT (Figure 1A,C–E). However, there were small but significant increases in bodyweight in both the T and T + EDP groups compared with the vehicle control group (Figure 1B). Although not significantly different from the control group, the T + EEQ group had a lower bodyweight than the T and T + EDP groups (*p* < 0.01 and *p* < 0.001, respectively) (Figure 1B). 

### 2.2. 17,18-EEQ or 19,20-EDP Combined with t-TUCB Improved Fasting Glucose and Serum TG Levels in Diet-Induced Obesity

**Figure 2:**
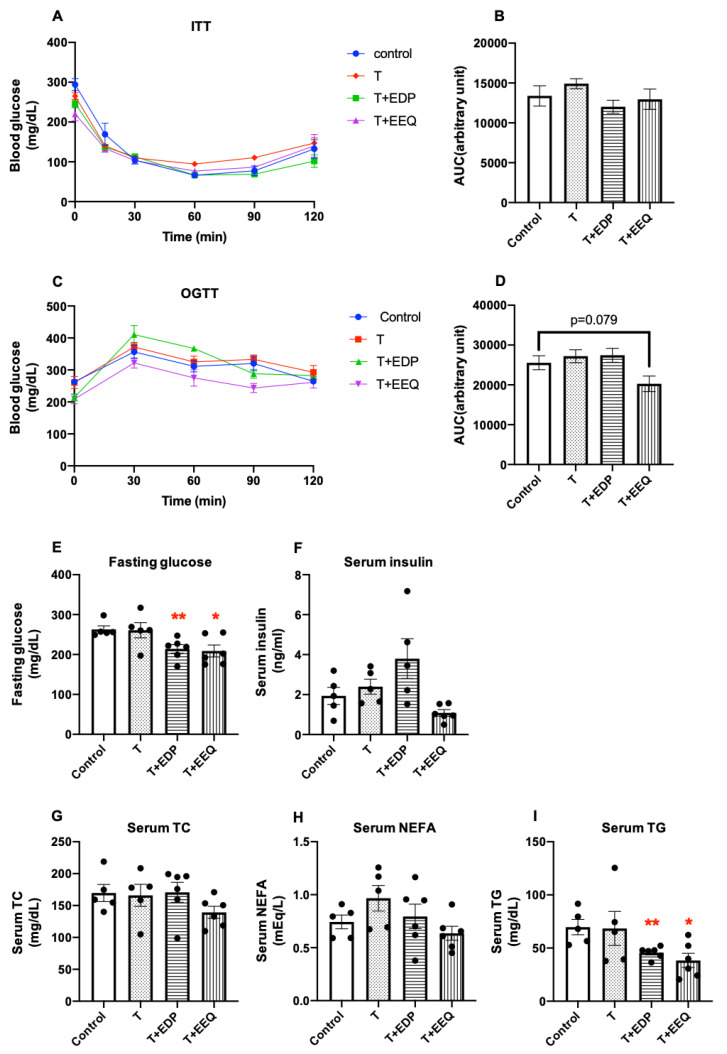
17,18-EEQ or 19,20-EDP combined with *t*-TUCB delivered via mini osmotic pump decreased the fasting glucose and serum TG levels in diet-induced obesity. Male C57BL/6J mice fed with a high-fat diet (45% kcal from fat) were treated with *t*-TUCB (3 mg/kg/day) alone or combined with 19,20-EDP (0.05 mg/kg/day) (T + EDP) or 17,18-EEQ (0.05 mg/kg/day) (T + EEQ) by osmotic minipump implantation for 6 weeks, as described. Insulin tolerance test and the area under the curve (AUC) (**A**,**B**), oral glucose tolerance test and the area under the curve (**C**,**D**), fasting glucose levels (**E**), serum insulin (**F**), serum TC (**G**), NEFA (**H**), and TG levels (**I**) after 6 weeks of implantation are shown. Data = the mean ± SEM (*n* = 5–6). *, ** = *p* < 0.05 and *p* < 0.01 compared to the controls, respectively.

To investigate the effects of sEH inhibition combined with 17,18-EEQ or 19,20-EDP on glucose metabolism, we performed insulin tolerance tests (ITT) and oral glucose tolerance tests (OGTT). Although there were no differences in the ITT among groups (Figure 2A,B), the T + EEQ group showed a trend of improvement in glucose tolerance tests (*p* = 0.079) (Figure 2C,D). Additionally, while there were no significant differences in the serum insulin levels among groups (Figure 2F), both the T + EEQ and T + EDP groups showed a significant improvement in fasting glucose compared to the control (208.8 ± 36.4 mg/d vs. 214 ± 10.8 mg/dL vs. 262.8 ± 8.9 mg/dL) (*p* < 0.05 and *p* < 0.01, respectively) (Figure 2E). 

We have previously shown that *t*-TUCB decreased the serum triglyceride (TG) levels in diet-induced obese mice [24]. To determine whether *t*-TUCB combined with 17,18-EEQ or 19,20-EDP can improve lipid metabolism in diet-induced obesity, we investigated the blood lipid parameters (Figure 2G–I). While the serum total cholesterol (TC) and non-esterified free fatty acids (NEFA) levels were unchanged in response to treatment with *t*-TUCB alone or combined with 17,18-EEQ or 19,20-EDP (Figure 2G,H), the serum TG levels were significantly decreased in the T + EDP (45.8 ± 2.2 mg/dL; *p* < 0.01) and T + EEQ (38.3 ± 6.7 mg/dL; *p* < 0.05) groups compared to the controls (69.7 ± 7.2 mg/dL) (Figure 2I).

### 2.3. 17,18-EEQ or 19,20-EDP Combined with t-TUCB Increased Thermogenesis in Diet-Induced Obesity

**Figure 3:**
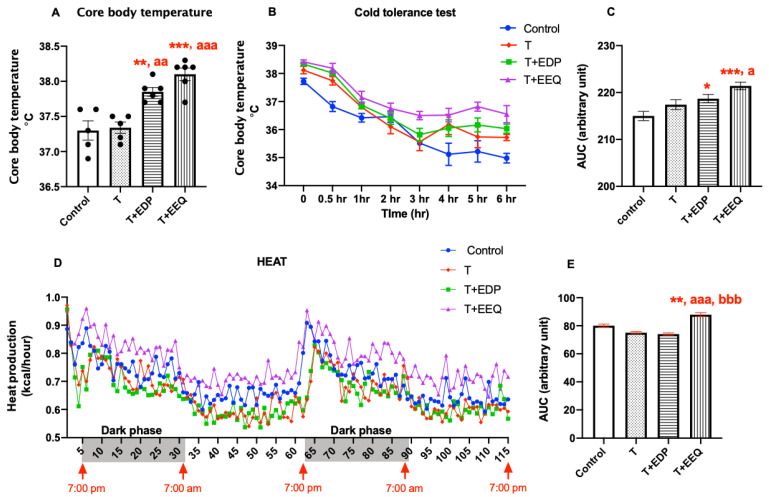
17,18-EEQ or 19,20-EDP combined with *t*-TUCB delivered via mini osmotic pump increased thermogenesis in diet-induced obesity. Male C57BL/6J mice fed a high-fat diet (45% kcal from fat) were treated with *t*-TUCB alone (3 mg/kg/day) or combined with 19,20-EDP (0.05 mg/kg/day) (T + EDP) or 17,18-EEQ (0.05 mg/kg/day) (T + EEQ) by osmotic minipump implantation for 6 weeks, as described. Core body temperature (**A**), cold tolerance test and the area under the curve (AUC) (**B**,**C**), and heat production and the area under the curve (AUC) (**D**,**E**) after 6 weeks of minipump implantation are shown. Data = the mean ± SEM (*n* = 5–6) (**A**–**C**,**E**), or only the mean (*n* = 5–6) (**D**). *, **, and *** = *p* < 0.05, *p* < 0.01, and *p* < 0.001 compared to the controls, respectively. ^a^, ^aa^, and ^aaa^ = *p* < 0.05, *p* < 0.01, and *p* < 0.001 compared to the T group, respectively. ^bbb^ = *p* < 0.001 compared to the T + EDP group.

To investigate whether the beneficial effects of 17,18-EEQ or 19,20-EDP combined with *t*-TUCB on metabolism are correlated with improved thermogenesis, we measured the core body temperature and performed a cold tolerance test and indirect calorimetry (Figure 3). These tests were performed at room temperature. Core body temperature was not changed by *t*-TUCB (37.3 ± 0.08 °C) but was significantly increased in the T + EDP (37.9 ± 0.06 °C; *p* < 0.01) and T + EEQ (38.1 ± 0.09 °C; *p* < 0.001) groups compared to the controls (37.3 ± 0.14 °C) (Figure 3A). In addition, both T + EDP and T + EEQ groups showed significantly higher core body temperature than the T group (*p* < 0.01 and *p* < 0.001, respectively). Consistently, both the T + EDP and T + EEQ groups showed significantly improved cold tolerance compared to the control (*p* < 0.05 and *p* < 0.001, respectively) (Figure 3B,C). The T + EEQ group also showed better cold tolerance than the T group (*p* < 0.05). Furthermore, heat production, measured by indirect calorimetry, was significantly higher in the T + EEQ group (*p* < 0.01) but not in the T and T + EDP groups compared to the control group (Figure 3D,E). 

### 2.4. 17,18-EEQ or 19,20-EDP Combined with t-TUCB Decreased Lipid Accumulation and Regulated Protein Expression of Thermogenic Genes in the iBAT of Diet-Induced Obesity

Since 17,18-EEQ or 19,20-EDP combined with *t*-TUCB alleviated some of the obesity-induced metabolic dysregulation and increased thermogenesis, we further investigated whether 17,18-EEQ or 19,20-EDP combined with *t*-TUCB modulated BAT activation, leading to the beneficial effects.

**Figure 4:**
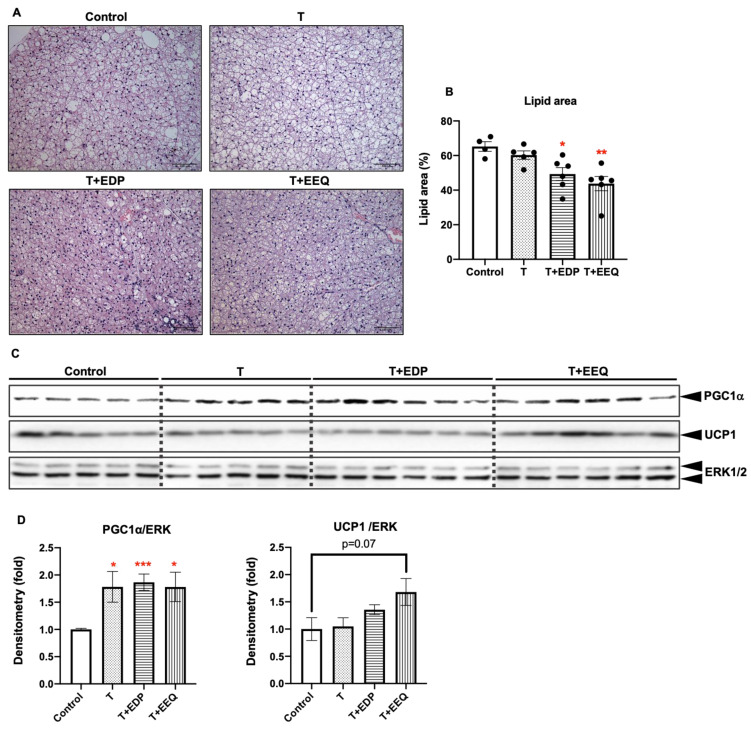
17,18-EEQ or 19,20-EDP combined with *t*-TUCB delivered via mini osmotic pump decreased the lipid accumulation and regulated thermogenic gene expression in the iBAT of diet-induced obesity. After termination, the iBAT tissue slides from mice were stained with hematoxylin and eosin (H&E; **A**), and the % area occupied by lipid from 2–3 fields per slide per mouse (*n* = 5) were analyzed using Image J software (**B**). Scale bars = 100 µm. Protein expression of PGC1α, UCP1, and the loading control ERK in the iBAT of mice in the control, T, T + EDP, and T + EEQ group (**C**), and their densitometry (**D**) are shown; Bar graphs show the normalized densitometry for PGC1α/ERK1/2 and UCP1/ERK1/2. Data = the mean ± SEM (*n* = 5–6). *, **, and *** = *p* < 0.05, *p* < 0.01, and *p* < 0.001 compared to the controls, respectively.

First, we examined changes in lipid accumulation in the iBAT by measuring the % area occupied by lipid from hematoxylin–eosin (H&E)-stained iBAT slides of each mouse (Figure 4A,B). *t*-TUCB alone did not change the lipid accumulation compared to the controls, but the lipid accumulation in the iBAT of the T + EDP and T + EEQ groups was significantly lower than the controls (*p* < 0.05 and *p* < 0.01, respectively). In comparison, we also investigated whether the local delivery of *n*-3 epoxides combined with *t*-TUCB to the iBAT could have effects on the subcutaneous white adipose tissue (sWAT). We found that the average adipocyte areas in the sWAT were significantly decreased in the T + EEQ group but not in the T or T + EDP groups (*p* < 0.05) (Supplemental Figure S2A).

Next, we analyzed the protein expression of thermogenic genes in the iBAT (Figure 4C,D). We found that the UCP1 protein expression in the iBAT was not changed in the T and T + EDP groups, but the T + EEQ group showed a trend of an increase in UCP1 protein expression compared to the controls (*p* = 0.07). On the other hand, PGC1α protein expression in the T, T + EDP, and T + EEQ groups was significantly increased compared to the control group (*p* < 0.05, *p* < 0.001, and *p* < 0.05, respectively). However, UCP1 protein expression was not readily detectable in the sWAT (data not shown). In contrast, PGC1α protein expression was significantly increased in the sWAT of the T + EEQ group compared to the controls (*p* < 0.05) (Supplemental Figure S2B,C).

### 2.5. 17,18-EEQ or 19,20-EDP Combined with t-TUCB Regulated Protein Expression of Genes Involved in Lipid Metabolism in the iBAT of Diet-Induced Obesity

**Figure 5:**
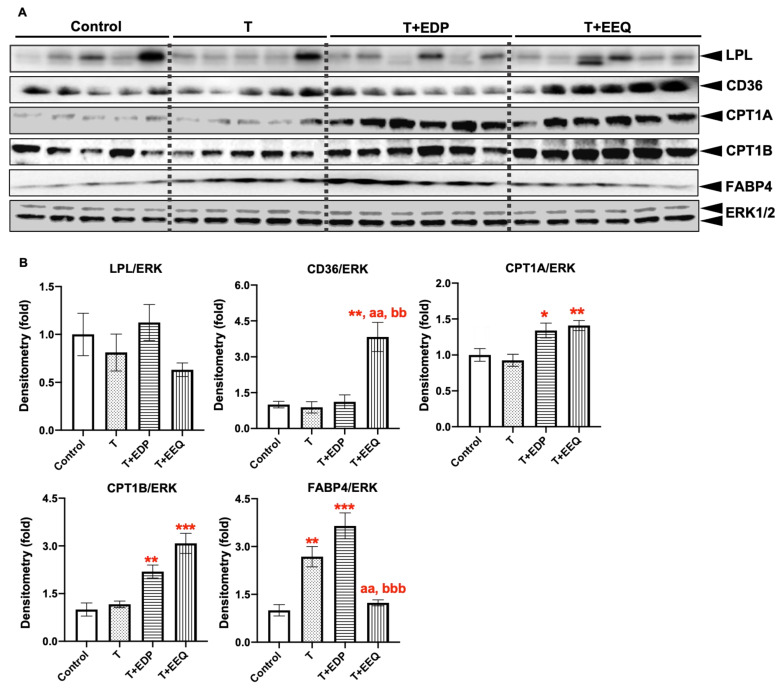
17,18-EEQ or 19,20-EDP combined with *t*-TUCB delivered via mini osmotic pump regulated the protein expression of lipid metabolic genes in the iBAT of diet-induced obesity. Protein expression of LPL, CD36, CPT1A, CPT1B, FABP4, and the loading control ERK in the iBAT of mice in the control, T, T + EDP, and T + EEQ groups (**A**), and their densitometry (**B**) are shown. Bar graphs show the normalized densitometry for LPL/ERK1/2, CD36/ERK1/2, CPT1A/ERK1/2, CPT1B/ERK1/2, and FABP4/ERK1/2. Data = the mean ± SEM (*n* = 5–6). *, **, and *** = *p* < 0.05, *p* < 0.01, and *p* < 0.001 compared to the control, respectively. ^aa^ = *p* < 0.01 compared to the T group. ^bb^ and ^bbb^ = *p* < 0.01 and *p* < 0.001 compared to the T + EDP group, respectively.

We have previously demonstrated that *t*-TUCB decreased the serum TG levels accompanied by increased protein expression of PLIN, a gene involved in lipid lipolysis, in the iBAT of diet-induced obese mice [24]. Consistently, we show that the T + EDP and T + EEQ groups had significantly lower serum TG levels in this study. Moreover, the lipid accumulation in the iBAT was significantly decreased by 19,20-EDP or 17,18-EEQ combined with *t*-TUCB. Therefore, we further investigated the protein expression of genes involved in lipid metabolism in the iBAT of diet-induced obesity (Figure 5).

First, protein expression of LPL, CD36, FABP4, CPT1A, and CPT1B, which are involved in fatty acid uptake, transport, binding, and oxidation in the iBAT, were analyzed by Western blot analysis (Figure 5). There were no significant changes in LPL expression in the iBAT among groups. In contrast, CD36 expression was not changed in both the T and T + EDP groups but was significantly increased in the T + EEQ group compared to the control, T, or T + EDP group (*p* < 0.01). Moreover, CPT1A and CPT1B protein expression were significantly increased in both the T + EDP (*p* < 0.05 for CPT1A and *p* < 0.01 for CPT1B) and T + EEQ groups (*p* < 0.01 for CPT1A and *p* < 0.001 for CPT1B), but not in the T group. In addition, FABP4 protein expression was significantly higher in both the T and T + EDP groups (*p* < 0.01 and *p* < 0.001, respectively), consistent with their increased bodyweights. In addition, there were no changes in CD36 protein expression among all three treated groups compared to the controls in the sWAT (Supplemental Figure S2B,C). No significant differences were observed in the FABP4 protein expression among groups in the sWAT (Supplemental Figure S2B,C).

Next, we examined the protein expression and phosphorylation of genes involved in lipolysis (Supplemental Figure S3). Consistent with previous results, PLIN expression was significantly increased in the iBAT of mice treated with *t*-TUCB (*p* < 0.05) alone or combined with 19,20-EDP (*p* < 0.001) or 17,18-EEQ (*p* < 0.05). In addition, phosphorylation of PLIN at S517 was significantly higher in the T, T + EDP, and T + EEQ groups compared to the controls (*p* < 0.05, *p* < 0.05, and *p* < 0.01, respectively). In addition, HSL protein expression and phosphorylation were differentially regulated in the T + EEQ group compared to the other groups (Supplemental Figure S3). There were no significant changes in ATGL protein expression in the iBAT among all groups (Supplemental Figure S3). In contrast, PLIN protein expression was significantly increased in the sWAT in the T + EEQ group (*p* < 0.05) (Supplemental Figure S2B,C).

### 2.6. 17,18-EEQ or 19,20-EDP Combined with t-TUCB Decreased Inflammatory Response in the iBAT of Diet-Induced Obesity

**Figure 6:**
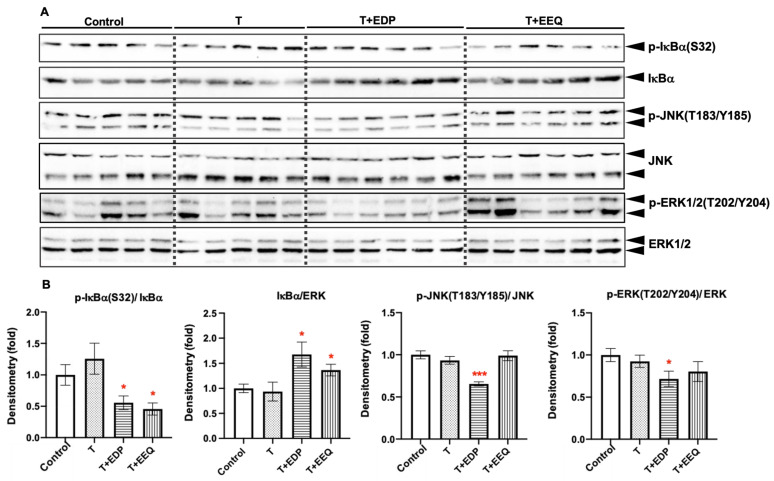
17,18-EEQ or 19,20-EDP combined with *t*-TUCB delivered via mini osmotic pump regulated the activation of inflammatory pathways in the iBAT of diet-induced obesity. Protein expression of p-IκBα(S32), IκBα, p-JNK(T183/Y185), JNK, p-ERK1/2(T202/Y204), and ERK1/2 in the iBAT of mice in the control, T, T + EDP, and T + EEQ groups (**A**), and their densitometry (**B**) are shown. Bar graphs show the normalized densitometry for p-IκBα(S32)/IκBα, IκBα, p-JNK(T183/Y185)/JNK, and p-ERK(T202/Y204)/ERK. Data = the mean ± SEM (*n* = 5–6). * and *** = *p* < 0.05 and *p* < 0.001 compared to the control, respectively.

It has been reported that the BAT content and activity declined in the obese animals partly because of the inflammatory microenvironment of the BAT caused by low-grade inflammation in obesity [25,26]. Targeting systematic and local inflammation could be a possible way to restore BAT function, thereby improving metabolic health [27]. As the parental PUFAs of 17,18-EEQ and 19,20-EDP, EPA and DHA have long been reported to regulate inflammatory processes in both rodents and humans [28,29]. Moreover, the anti-inflammatory roles of 17,18-EEQ or 19,20-EDP have been revealed in multiple disease models [30]. Therefore, we analyzed whether 17,18-EEQ or 19,20-EDP combined with *t*-TUCB can suppress inflammatory pathways in the iBAT of diet-induced obese mice, thereby improving the iBAT activity (Figure 6).

As the hallmarks of NFκB activation, the phosphorylation of IκBα at S32 was significantly lower, and the IκBα abundance was significantly higher in both the T + EDP and T + EEQ groups than the control group (*p* < 0.05). However, the phosphorylation of JNK and ERK, the hallmarks of activation of the inflammatory MAPK pathways, were only decreased in the T + EDP group compared to the control group (*p* < 0.05).

## 3. Discussion

BAT has emerged as a promising target to combat obesity and its associated metabolic disorders due to its role in nonshivering thermogenesis [31,32,33]. In the current study, we have demonstrated that the fatty acid epoxide 17,18-EEQ or 19,20-EDP combined with *t*-TUCB via mini osmotic pump delivery significantly decreased the fasting glucose and serum TG levels while it increased the core body temperature, as well as improved the cold tolerance and thermogenesis in diet-induced obesity. In addition, 17,18-EEQ or 19,20-EDP combined with *t*-TUCB reduced the lipid accumulation in the iBAT, which was accompanied by enhanced protein expression of genes involved in thermogenesis and lipid metabolism and reduced the inflammatory response in the iBAT. In contrast, the average adipocyte areas in the sWAT were significantly decreased in the T + EEQ group, which was accompanied by increases in PGC1*α* and PLIN protein expression in the sWAT in the T + EEQ group. 

### 3.1. 17,18-EEQ and 19,20-EDP Contribute to the Beneficial Effects of EPA and Fish Oil

As the precursors of EEQs and EDPs, EPA and fish oil (known to be enriched with both DHA and EPA) have long been reported to improve obesity and its associated metabolic disorders, at least in part by regulating the energy expenditure and/or thermogenesis in rodents [34,35,36,37]. Many of the animal studies with fish oil [38,39,40] showed increased energy expenditure and/or thermogenesis and improvement in glucose and lipid metabolism with concurrent increases in thermogenic Ucp1 and/or Pgc1α expression in the BAT; however, increased energy expenditure and improvement in metabolism without significant increases in Ucp1 and/or Pgc1α expression have also been reported [41], possibly due to the differences in study designs, fish oil source and composition, and the dose and duration of the treatments. Mice studies with pure EPA showed dose-dependent increases in energy expenditure [42] and increased UCP1 protein expression at the highest dose tested [43]. However, whether the downstream epoxidized metabolites of EPA and DHA are at least partially responsible for the beneficial effects remains elusive. Our study has shed new light on the mechanisms underlying the anti-obesity effects of *n*-3 PUFAs by demonstrating that their downstream epoxide metabolites, 17,18-EEQ and 19,20-EDP, when combined with *t*-TUCB, had similar regulatory effects on energy expenditure, thermogenesis, and glucose and lipid metabolism accompanied by changes in gene expression of Pgc1α and Ucp1. Our results suggest that 17,18-EEQ and 19,20-EDP may at least in part contribute to the beneficial effects reported for EPA and fish oil. 

### 3.2. 17,18-EEQ Is More Potent in Increasing Thermogenesis and Improving Metabolism in Diet-Induced Obesity

Interestingly, differential effects among the three treatment groups are noted in our study. Even though both the T + EEQ and T + EDP group showed beneficial effects in thermogenesis and metabolic biomarkers (i.e., increased core body temperature and cold tolerance and improved fasting glucose and serum TG levels, and less lipid accumulation in the BAT), which are not observed in the T group, it is clear that T + EEQ has more potent effects than the T + EDP group. Moreover, significant increases in heat production were only found in the T + EEQ group. Limited studies comparing the effects of EPA and DHA have suggested that EPA seemed to be more potent than DHA as a thermogenic stimulus [34]. To explore the molecular mechanisms by which the 17,18-EEQ or 19,20-EDP combined with the sEH inhibitor showed differential effects, we further analyzed the protein expression of the genes involved in lipid metabolism and inflammatory pathways in the BAT. 

Upon cold stimulation, BAT consumes free fatty acids primarily derived from the lipolysis of intracellular lipid droplets [44,45], which need to be replenished by the uptake of circulating TG-enriched lipoproteins [9,46]. In addition, fatty acid oxidation (FAO) in mitochondria is enhanced to meet the high energetic demands [47,48]. Therefore, activated BAT shows increases in FAO and enhances TG clearance from circulation [9]. We found that the serum TG levels and lipid accumulation in the iBAT were decreased by 17,18-EEQ or 19,20-EDP combined with *t*-TUCB. Therefore, we further investigated the protein expression of the genes responsible for lipolysis, FAO, and fatty acids uptake in the BAT. 

In adaptive thermogenesis, lipolysis in brown adipocytes was mediated by PLIN, a lipid-coating protein whose phosphorylation promotes lipase activity in response to β-adrenergic stimulation [49]. We found that in all three treatment groups, PLIN protein abundance and phosphorylation of PLIN at S517 were significantly increased, indicating enhanced intracellular lipolysis for all three treatment groups, consistent with our previous report [24]. In addition, we observed that HSL protein expression and phosphorylation were differentially regulated in the T + EEQ group compared to the other groups. HSL hydrolyzes diacylglycerol and produces monoacylglycerol, which is further hydrolyzed by the monoacylglycerol lipase [50]. Phosphorylation of HSL has been shown to affect HSL-mediated lipolysis [51,52,53]. The effects of 17,18-EEQ on lipolysis need to be determined in future studies.

As the rate-limiting enzyme in FAO, the CPT1 family has three isoforms, including CPT1A, B, and C, distributed differently among tissues [54,55,56]. Although both CPT1A and CPT1B are expressed in the BAT of mice and rats, CPT1B has a much higher expression than CPT1A [54,55]. Specific CPT1 inhibition by 2-tetradecylglycidic acid (McN-3802) decreased palmitic acid-induced mitochondrial respiration in murine primary brown adipocytes [57]. While Cpt1b^−/−^ mice were embryonically lethal, more than half of the Cpt1^+/−^ mice developed fatal hypothermia after a prolonged cold exposure compared to 21% of Cpt1b^+/+^ mice [58]. Moreover, overexpressing an active mutant form of CPT1A (insensitive to malonyl-CoA inhibition) in fully matured rat brown adipocytes significantly enhanced FAO, lipolysis, and mitochondria activity, which in turn inhibited the palmitate-induced triglyceride accumulation [59]. Our results show that both the T + EEQ and T + EDP groups had significantly increased CPT1A and CPT1B protein expression in the BAT, suggesting that these epoxides combined with *t*-TUCB, but not the *t*-TUCB alone, may enhance FAO, thereby promoting BAT activity in diet-induced obesity.

LPL is a critical lipase that is involved in TG-enriched lipoprotein hydrolysis for the uptake of free fatty acids into the cells [60]. Short-term cold exposure [61] and cold-induced lipokine [62] increase free fatty acids uptake in the BAT by regulating the LPL activity and expression. However, we did not find changes in LPL protein expression among the different treatment groups, which were housed under room temperature. CD36 is a cell surface protein responsible for fatty acid uptake from circulation and was also upregulated in the BAT by the cold temperature and contributed to cold-induced TG clearance [61]. CD36^−/−^ mice showed decreases in fatty acid uptake and thermogenic gene expression in the BAT after cold exposure, leading to impaired cold tolerance [63]. In addition, CD36 is indispensable for the BAT thermogenic function by mediating coenzyme Q uptake from circulation for mitochondria respiration [64]. Interestingly, we found that CD36 protein expression was only upregulated in the T + EEQ group compared to other treated groups, consistent with the increased heat production in the T + EEQ group. Our results suggest that CD36 may be specifically regulated by 17,18-EEQ but not by 19,20-EDP, which partially contributes to more potent thermogenic effects observed in the T + EEQ group. The molecular mechanisms by which 17,18-EEQ upregulates CD36 need further investigation. 

In comparison, the average area of adipocytes in the sWAT was significantly decreased in the T + EEQ group, which is associated with increases in PGC1*α* and PLIN protein expression in the sWAT of the T + EEQ group. The results suggest that the decrease in the average areas of adipocytes in the sWAT in the T + EEQ group may be due to enhanced mitochondrial biogenesis and lipolysis induced by increased protein expression of PGC1*α* and PLIN, respectively, by the T + EEQ. Further studies are needed to investigate the effects of n-3 epoxides on WAT. 

### 3.3. Both 17,18-EEQ or 19,20-EDP Suppress NFκB Activation in the BAT of Diet-Induced Obesity

Obesity is associated with low-grade chronic inflammation, leading to impaired BAT activity, which contributes to the development of insulin resistance and type 2 diabetes [25,26]. Therefore, targeting the inflammatory pathways may reverse the BAT dysfunction and prevent the development of metabolic disorders [27]. We found that the fasting glucose levels were significantly decreased by both 17,18-EEQ and 19,20-EDP combined with *t*-TUCB, and there was a trend of an improvement in glucose tolerance in the T + EEQ group. Therefore, we further investigated the hallmarks of activation for inflammatory pathways in brown adipocytes [65]. We found that while activation of the JNK and EKR pathways were suppressed by T + EDP, NF*κ*B activation was significantly suppressed by both epoxides combined with *t*-TUCB. In contrast, *t*-TUCB alone did not suppress any of these inflammatory pathways under our study conditions. These results suggest anti-inflammatory effects of 17,18-EEQ and 19,20-EDP, which may contribute to the improvement of glucose metabolism and thermogenesis in diet-induced obesity.

Limited studies have been reported on the anti-inflammatory effects of 17,18-EEQ and 19,20-EDP on adipose tissue in diet-induced obesity [23,66]. In fat-1 mice with enriched endogenous n-3 PUFA by transgenic expression of n-3 desaturase, 17,18-EEQ and 19,20-EDP were significantly increased in the liver and eWAT, accompanied by deceased macrophage infiltration and reduced pro-inflammatory markers in the eWAT compare to wild type controls [23]. Moreover, injection of 17,18-EEQ combined with two metabolites of EPA from lipoxygenase (5-HEPE and 9-HEPE) for 4 days significantly reduced the inflammatory response in the adipose tissue of high-fat diet-induced obese mice [66]. Our results further suggest anti-inflammatory effects of 17,18-EEQ and 19,20-EDP on the BAT in diet-induced obesity. However, due to the limited amount of tissue samples, we were not able to analyze further the downstream mechanisms involved in glucose metabolism, such as GLUT4 translocation, in the BAT. Future studies on how 17,18-EEQ and 19,20-EDP improve glucose metabolism through suppression of BAT inflammation are warranted. 

### 3.4. Limitations

There are a few limitations in our studies. We only studied male C57BL/6J mice. Females of the C57BL/6J mice are not sensitive to a high-fat diet (our unpublished results) and may respond differently to 17,18-EEQ and 19,20-EDP. Future studies are needed to examine the effects of these epoxides on obesity and metabolic biomarkers in female mice. In addition, sEH ablation and an n-6 epoxide analog have been reported to induce browning in the WAT [67] and 3T3-L1 adipocytes [68]. However, consistent with our previous study [24], we did not find browning of sWAT by n-3 epoxides combined with *t*-TUCB in the mice housed at 22–23 °C. Although we delivered 17,18-EEQ, 19,20-EDP, and *t*-TUCB locally to the iBAT, we found the average areas of adipocytes of the sWAT were significantly decreased in the T + EEQ group accompanied by increases in PGC1*α* and PLIN protein expression, suggesting systemic effects of T + EEQ. Therefore, it is possible that these epoxides may improve systemic metabolism through modulating the activities of other metabolic active tissues, such as WAT and muscle. Further studies are needed to examine the effects of 17,18-EEQ or 19,20-EDP combined with sEH inhibitor on WAT and muscle. Lastly, the specific effects of 17,18-EEQ and 19,20-EDP on the BAT were extrapolated by comparing their biological effects when combined with *t*-TUCB with the effects by *t*-TUCB alone due to the quick metabolism of epoxides by the sEH. Future studies are needed to confirm the effects of 17,18-EEQ and 19,20-EDP on the BAT, using either sEH knockout (KO) mice or stable epoxide analogs. 

### 3.5. Summary

Overall, the results demonstrate that local delivery of 17,18-EEQ or 19,20-EDP combined with *t*-TUCB by osmotic pump to iBAT may promote BAT activity by regulating the protein expression of genes involved in mitochondrial biogenesis, lipolysis, fatty acid oxidation, and inflammatory pathways, leading to increased thermogenesis and improvement of glucose and lipid metabolism in diet-induced obesity. 17,18-EEQ may be more potent than 19,20-EDP in enhancing thermogenesis by upregulating a more comprehensive set of genes involved in lipid metabolism, including CD36, in the BAT. Future studies are needed to confirm the beneficial effects of these epoxides on the BAT using either sEH KO mice or stable epoxide analogs.

## 4. Materials and Methods

### 4.1. Reagents 

Antibodies used in the study are shown in the following table.

### 4.2. Animal Study

All animal procedures were approved by the Institutional Animal Care and Use Committee at the University of Tennessee, Knoxville (protocol# 2587, approved 18 February 2018). Mice were housed individually at 22–23 °C with a 12-h light/dark cycle. Three-week-old male C57BL/6J mice were purchased from the Jackson Laboratory and were fed a regular chow diet (14% kcal from fat, 8604) (Teklad Rodent Diet, ENVIGO) for 5 weeks. At 8 weeks old, 10 mice were randomly selected as the non-implantation controls. They were fed a low-fat diet (10% kcal from fat, D12450H) or a high-fat diet (45% kcal from fat, D12451) (Research Diets) for 6 weeks. At the same time, the rest of the mice were randomly divided into 4 groups (*n* = 5–6 per group) and were fed the same high-fat diet (45% kcal from fat, D12451) (Research Diets). In addition, these four groups of mice were surgically implanted with Alzet osmotic minipumps (model 2006) (DURECT Corporation, Cupertino, CA, USA) filled with the following: mixed solvent (the vehicle control), *t*-TUCB alone (T), or *t*-TUCB combined with 19,20-EDP (T + EDP) or 17,18-EEQ (T + EEQ). All pumps were implanted into the subcutaneous compartment from an interscapular incision nearby the iBAT. *t*-TUCB, 19,20-EDP, and 17,18-EEQ were dissolved in a mixed solvent (25% DMSO in polyethylene glycol 300 (PEG 300)), as previously described [69]. The deliver rate was 3 mg/kg/day for *t*-TUCB, and 0.05 mg/kg/day for 19,20-EDP and 17,18-EEQ, respectively. The maximal delivery duration was 6 weeks by design for the model. Bodyweight and food intake were measured every week. Insulin and glucose tolerance tests, indirect calorimetry, and cold tolerance tests were performed as previously described [24]. The animals were then terminated at room temperature. Whole blood was collected by cardiac puncture under anesthesia. Subcutaneous WAT, epididymal WAT, interscapular BAT, liver, and gastrocnemius muscle were collected and weighed. A small piece of tissue samples was fixed for histopathological analysis and the rest of the tissue samples were snap-frozen in liquid nitrogen and then stored at −80 °C until analysis.

### 4.3. Blood Biochemical Analysis 

The Mouse Glucose Assay kit purchased from Crystal Chem (Downers Grove, IL, USA) was used to measure the plasma glucose levels. An Ultra-Sensitive Mouse Insulin ELISA kit (Crystal Chem) was used to analyze the serum insulin levels. The serum lipid parameters, including serum triglycerides (TG), non-esterified fatty acids (NEFA), and cholesterol, were determined by respective tests from Wako Diagnostics (FUJIFILM Wako Diagnostics, Mountain View, CA, USA). All tests were performed according to the manufacturers’ instructions.

### 4.4. Western Blot Analysis

Tissue samples were homogenized by an electric homogenizer in 1xRIPA lysis buffer (Cell Signaling, Danvers, MA, USA) and then centrifuged for 15 min at 12,000 rpm at 4 °C to obtain the protein samples. A BCA assay kit (Thermo Scientific, Waltham, MA, USA) was used to determine the protein concentrations. Protein samples (1–45 µg per lane) were separated on a 12% SDS-PAGE and then transferred to polyvinylidene difluoride (PVDF) membranes purchased from Bio-Rad (Hercules, CA, USA) in the transfer buffer (25 mM Tris base, 190mM glycine, 20% methanol) at 25 volts for 20 h. Then the membranes were blocked in TBST buffer (20 mM Tris Base, 137 mM NaCl, and 0.1% Tween 20 (pH 7.4)) with 5% nonfat milk for 1 h at room temperature. Next, membranes were immunoblotted with the indicated primary antibodies (Table 1) against the proteins of interest at 4 °C overnight at a 1:1000 dilution (at 1:2000 dilution for UCP1 antibody, 1:500 dilution for CD36 and HSL antibodies). After a 10 min wash in TBST buffer, repeated 3 times, the membranes were incubated with secondary antibodies (horseradish peroxidase conjugated) at a 1:4000 dilution for 1 h. Bands of the protein of interest were visualized by Pierce ECL Western Blotting Substrate or SuperSignal West Pico Chemiluminescent Substrate (Thermo Scientific, Pittsburgh, PA, USA). To facilitate detection of some proteins of interest, two gels were run and detected at the same time to accommodate all samples and/or large sample volumes. Densitometry of the bands was quantified using the ChemiDoc XRS+ system with Image Lab software (Bio-Rad, Hercules, CA, USA) or Image J software (an open-source from NIH). 

### 4.5. BAT Lipid Accumulation and sWAT Adipocyte Area Determination 

The iBAT and sWAT from each mouse were isolated. A small piece was fixed in 10% neutral-buffered formalin and then processed for hematoxylin and eosin staining at the University of Tennessee College of Veterinary Medicine Diagnostic Laboratory Service. Nikon Eclipse E-600 microscopy was used to take pictures from 2–4 fields of each slide of iBAT or sWAT from each mouse. The % area occupied by lipid or adipocyte areas from 2–3 fields per slide per mouse was then quantified by the Image J software. 

### 4.6. Statistical Analysis

Prism 8 (GraphPad Software, San Diego, CA, USA) was used to conduct the statistical analysis. A two-tailed Student’s *t*-test was performed to determine the differences in group means among the treatment groups and the control group. For the glucose tolerance, insulin tolerance, cold tolerance tests, and heat production, areas under the curve (AUC) were calculated using Prism 8, and a two-tailed Student’s *t*-test was used to compare the differences between the treatment and the control group. The level of significance was set at *p* < 0.05.

## Figures and Tables

**Table 1:** Antibodies Used in the Study.

Antibodies	Company	Catalog Number
Anti-ATGL	Cell Signaling Technology (Danvers, MA, USA)	2439
Anti-CD36	Novus Biologicals (Centennial, CO, USA)	NB400-144
Anti-CPT1A	Cell Signaling Technology (Danvers, MA, USA)	12252S
Anti-CPT1B	Proteintech North America (Rosemont, IL, USA)	22170-1-AP
Anti-ERK1/2	Cell Signaling Technology (Danvers, MA, USA)	4695
Anti-FABP4	Cell Signaling Technology (Danvers, MA, USA)	2120S
Anti-IκBα	Cell Signaling Technology (Danvers, MA, USA)	9242S
Anti-JNK	Cell Signaling Technology (Danvers, MA, USA)	9252S
Anti-LPL	Santa Cruz Biotechnology (Dallas, TX, USA)	SC-373759
Anti-PGC1α	Millipore (Temecula, CA, USA)	AB3242
Anti-phospho-ERK(T202/Y204)	Cell Signaling Technology (Danvers, MA, USA)	4370S
Anti-phospho-IκBα	Cell Signaling Technology (Danvers, MA, USA)	2859S
Anti-phospho-JNK(T183/Y185)	Cell Signaling Technology (Danvers, MA, USA)	9251S
Anti-phospho-PLIN (S517)	Vala Sciences (San Diego, CA, USA)	4856
Anti-UCP1	Sigma Aldrich (St. Louis, MO, USA)	U6382
Lipolysis Activation Antibody Sampler Kit (Antibodies for phospho-HSL (Ser 563, Ser 565, and Ser 660), HSL, and PLIN)	Cell Signaling Technology (Danvers, MA, USA)	8334

## Supplementary Materials

Supplementary materials are available online at https://www.mdpi.com/article/10.3390/ijms22158267/s1.

## Abbreviations

ATGL	adipose triglyceride lipase
BAT	brown adipose tissue
CD36	cluster of differentiation 36
CPT1	carnitine palmitoyltransferase 1
CYP450	cytochrome P-450
DHA	docosahexaenoic acid
EDP	epoxydocosapentaenoic acid
EEQ	epoxyeicosatetraenoic acid
EPA	eicosapentaenoic acid
EpFA	epoxy fatty acids
ERK	extracellular signal-regulated kinases
FABP4	Fatty acids bind protein 4
FAO	Fatty acid oxidation
GTT	glucose tolerance test
HEPE	hydroxyeicosapentaenoic acid
HSL	hormone sensitive lipase
IκBα	nuclear factor of kappa light polypeptide gene enhancer in B-cells inhibitor, alpha
ITT	insulin tolerance test
JNK	c-Jun N-terminal Kinase
LPL	lipoprotein lipase
MAPK	mitogen-activated protein kinase
NEFA	non-esterified fatty acids
NF-κB	nuclear factor kappa B
PGC1α	peroxisome proliferator activator receptor coactivator 1-α
PLIN	perilipin
sEH	soluble epoxide hydrolase
TG	triglyceride
TC	total cholesterol
*t*-TUCB	trans-4-{4-[3-(4-trifluoromethoxy-phenyl)-ureido]-cyclohexyloxy-benzoic acid
UCP1	uncoupling protein 1
WAT	white adipose tissue
