# Supplementary material for: Differential Effects of 17,18-EEQ and 19,20-EDP Combined with Soluble Epoxide Hydrolase Inhibitor t-TUCB on Diet-Induced Obesity in Mice
Source: Int J Mol Sci. 2021 Jul 31;22(15):8267. doi: 10.3390/ijms22158267 (PMC8347952; doi:10.3390/ijms22158267)
Supplement: Supplementary file 1 [file ijms-22-08267-s001.zip › ijms-1322466-supplementary.pdf]

International Journal of
Molecular Sciences

MDPI

Differential Effects of 17,18-EEQ and 19,20-EDP Combined with Soluble Epoxide Hydrolase Inhibitor *t*-TUCB on Diet-induced Obesity In Mice

Yang Yang[1,] Xinyun Xu[1], Haoying Wu[1], Jun Yang[2], Jiangang Chen[3], Christophe Morisseau[2], Bruce D. Hammock[2], Ahmed Bettaieb[1,4,] and Ling Zhao[1,*]

Supplemental Figures and Tables

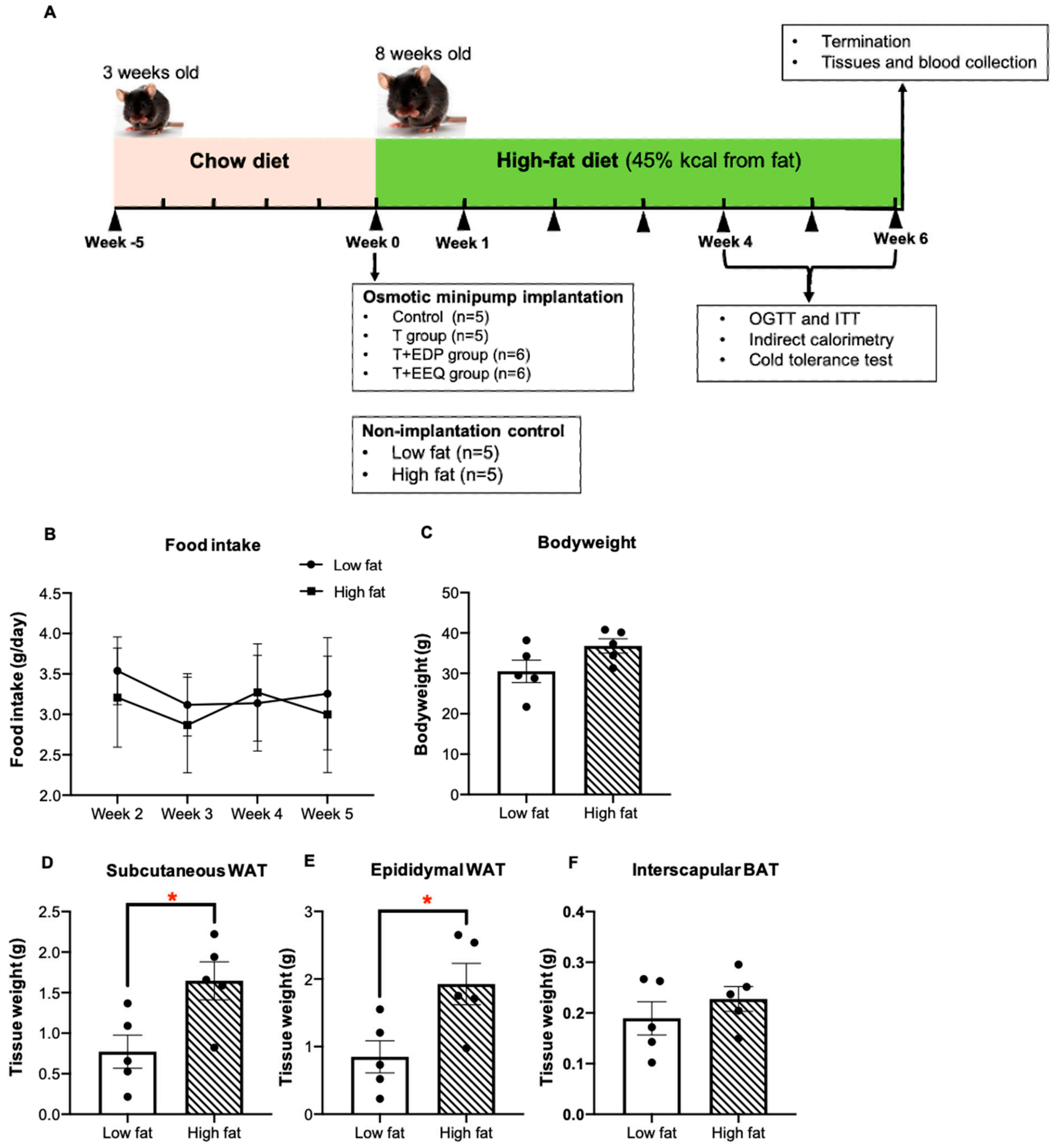

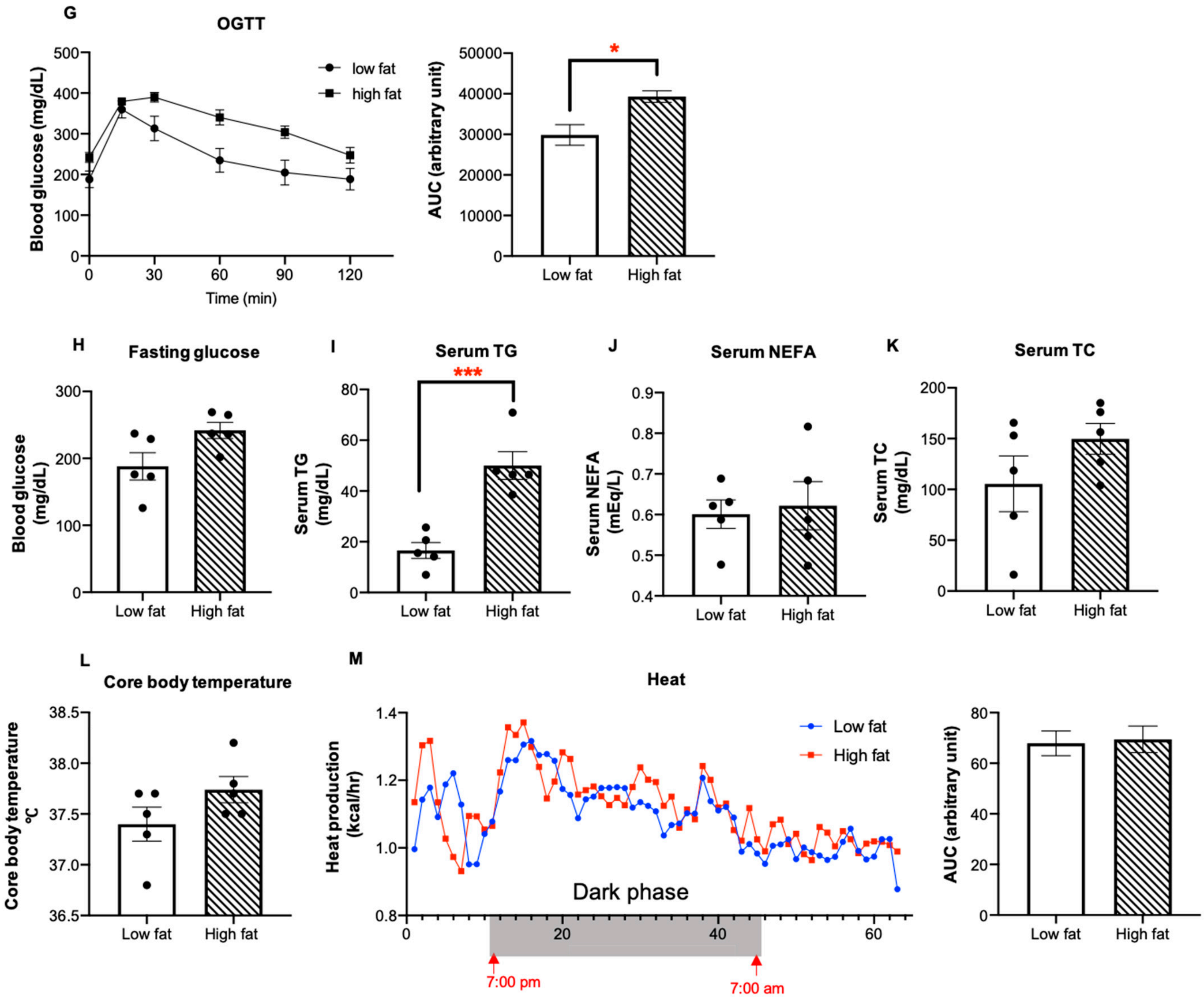

Supplemental Figure S1. The effects of the high-fat diet on food intake, bodyweight, fat pad weight, glucose tolerance, fasting glucose, serum TG, NEFA, and TC, core body temperature, and heat production in C57BL/6J mice.

Male C57BL/6J mice (8 weeks of age) were fed either a low-fat or a high-fat diet for 6 weeks as non-implantation controls. Bodyweight, food intake, fasting glucose were recorded, and the oral glucose tolerance test, core body temperature, and indirect calorimetry were performed as described. (A) Schematic study design. (B) Changes of food intakes; (C-F) Bodyweight (C) and various fat pad weights at termination (D-F); (G) Oral glucose tolerance test and the area under the curve (AUC); (H-K) Fasting blood glucose (H), serum TG (I), serum NEFA (J) and serum TC (K) levels at termination; (L) Core body temperature at termination; (M) Heat production and the area under the curve (AUC) are shown. Data = the mean±SEM (n=5). *, ***, p<0.05 and p<0.001 compared to the low-fat group, respectively.

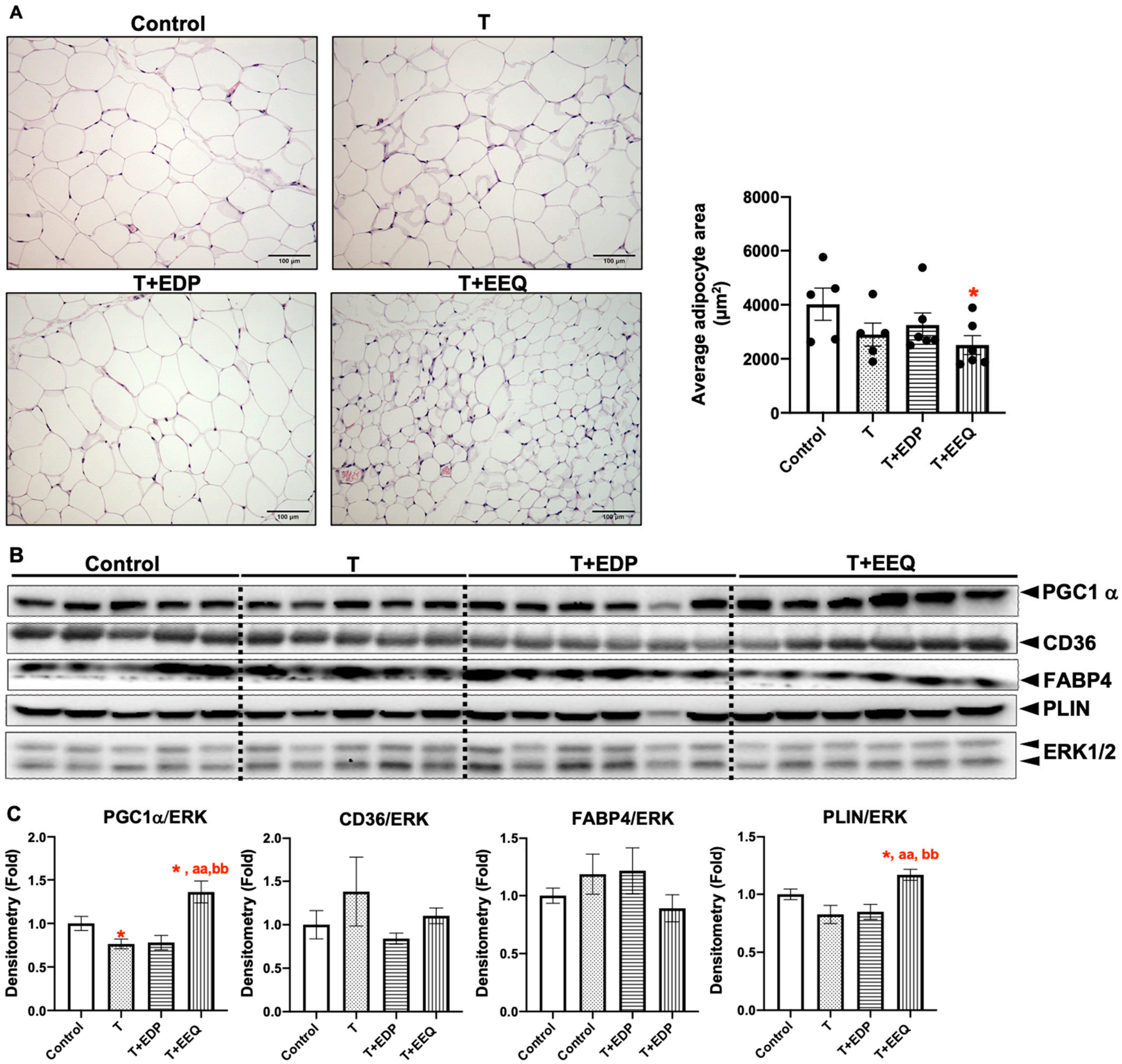

Supplemental Figure S2. 17,18-EEQ combined with *t*-TUCB delivered via mini osmotic pump decreased the average adipocyte areas and regulated protein expression in the sWAT of C57BL/6J mice in diet-induced obesity.

After termination, the sWAT slides from mice were stained with hematoxylin and eosin (H&E), and the adipocyte areas from 3-4 fields per slide per mouse (n=5) were analyzed using Image J software (A). Scale bars=100 µm. Protein expression of PGC1α, CD36, FABP4, PLIN and the loading control ERK1/2 in the sWAT of mice in the control, T, T+EDP, and T+EEQ groups (B) and their densitometry (C) are shown; Bar graphs show normalized densitometry for PGC1α/ERK1/2, CD36/ERK1/2, FABP4/ERK1/2, and PLIN/ERK1/2. Data = the mean±SEM (n=5-6). *, $p<0.05$ compared to the controls. aa, $p<0.01$ compared to the T group. bb, $p<0.01$ compared to the T+EDP group, respectively.

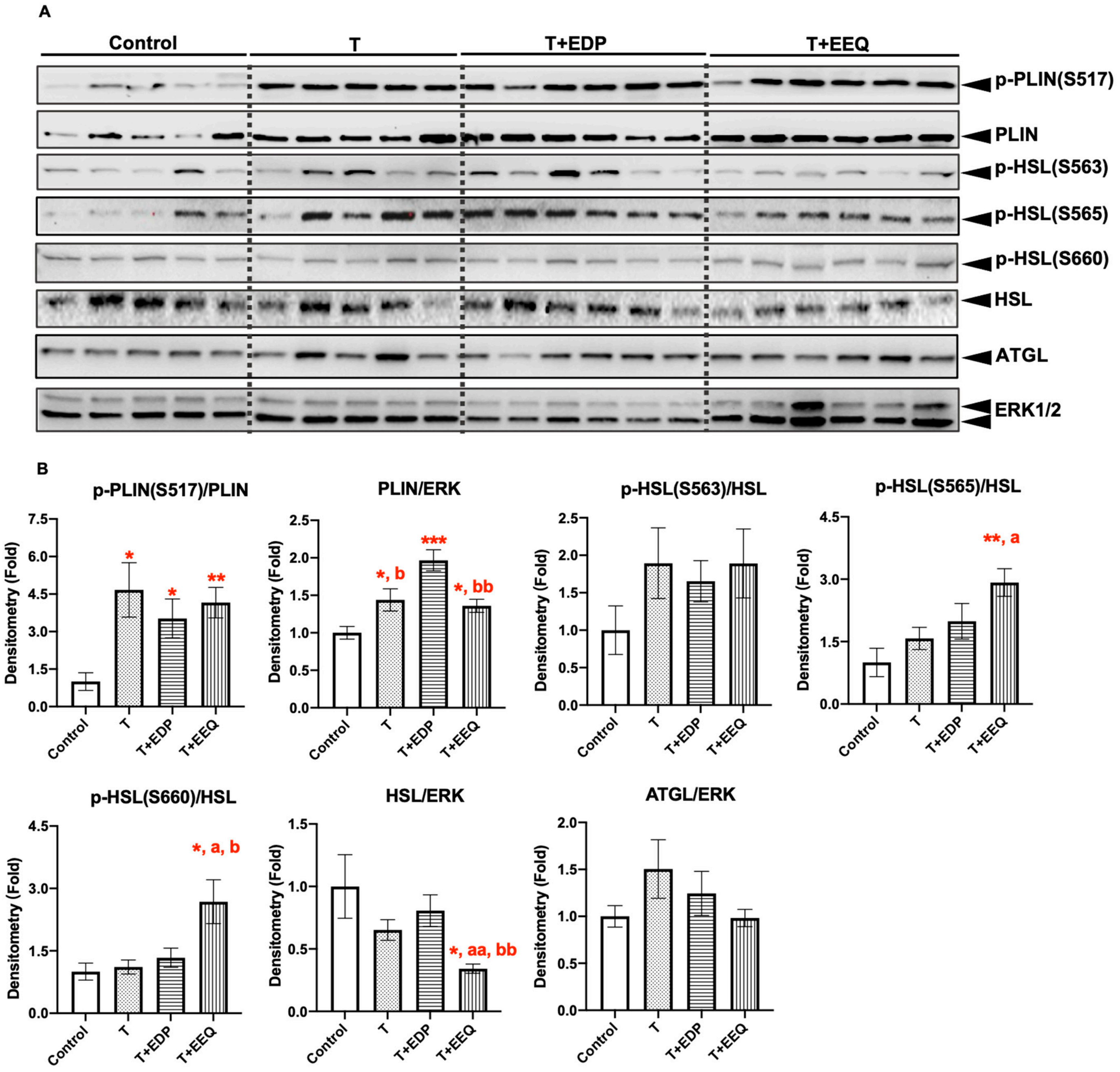

Supplemental Figure S3. 17,18-EEQ or 19,20-EDP combined with *t*-TUCB delivered via mini osmotic pump regulated protein expression of genes involved in lipolysis in the iBAT of C57BL/6J mice in diet-induced obesity.

Protein expression of PLIN, HSL, ATGL, the phosphorylation of PLIN and HSL, and the loading control ERK1/2 in the iBAT of mice in the control, T, T+EDP, and T+EEQ groups (A) and their densitometry (B) are shown; Bar graphs show normalized densitometry for p-PLIN(S517)/PLIN, PLIN/ERK1/2, p-HSL(S563)/HSL, p-HSL(S565)/HSL, p-HSL(S660)/HSL, HSL/ERK1/2, and ATGL/ERK1/2. Data = the mean±SEM (n=5-6). *, **, ***, $p<0.05$, $p<0.01$, and $p<0.001$ compared to the controls, respectively. a and aa, $p<0.05$, and $p<0.01$ compared to the T group, respectively. b and bb, $p<0.05$ and $p<0.01$ compared to the T+EDP group, respectively.
